# Experimental Evaluation of Compressive Properties of Early-Age Mortar and Concrete Hollow-Block Masonry Prisms within Construction Stages

**DOI:** 10.3390/ma17163970

**Published:** 2024-08-09

**Authors:** Ali Abasi, Bennett Banting, Ayan Sadhu

**Affiliations:** 1 Civil and Environmental Engineering Department, Western University, London, ON N6A 3K7, Canada; 2 Canada Masonry Design Centre, Ottawa, ON K1P 5K8, Canada

**Keywords:** early-age mortar, early-age masonry, compressive properties, masonry prisms, curing time

## Abstract

Early-age masonry structures require temporary support until they achieve full strength. Nevertheless, there is a limited understanding of the properties of freshly laid masonry and the design of newly constructed, unsupported masonry walls. This situation has led to numerous instances of structural damage and injuries to workers, prompting conservative construction bracing techniques. This paper presents comprehensive experimental studies on early-age mortar cubes and masonry prisms to assess the effects of curing time on the compressive properties of masonry assemblies, which is necessary for the design of temporary bracing. The change in modulus of elasticity and compressive strength of masonry prisms and mortar with curing time has been experimentally assessed. The results indicate that the compressive strength of freshly cast mortar cubes is relatively insignificant until approximately 24 h after construction, when it was observed to increase logarithmically. Regarding the performance perspective, the compressive strength of early-age masonry prisms is inconsiderable, less than 15% of full strength during the first day after construction. By contrast, regarding the life safety perspective, the compressive properties of a mortar joint within a masonry assembly (which is of more practical interest) appear to have no effect on the failure strength of concrete masonry prisms over the range of ages tested. The failure modes of the early-age mortar cubes and early-age masonry prism samples depend on the curing time, and different failure modes occurred before and after the start of the primary hydration phase, which is 20.8 h after construction. It is anticipated that the proposed research will provide valuable material properties leading to efficient design of control devices (e.g., temporary bracing) and improved guidelines for concrete-block masonry construction.

## 1. Introduction

Over the past few decades, significant advancements have been made in construction materials [1]. Nevertheless, masonry remains recognized as one of the most cost-effective options for large-scale construction worldwide [2], including in North America [3]. The properties of masonry, such as the minimum specified compressive strength of masonry (*f′_m_*), flexural tensile strength, and durability, are contingent upon the characteristics of individual components like mortar, grout, units, and reinforcement, as well as the interactions between these materials during construction [4]. The complexity of masonry, as a heterogeneous material, adds to the intricacies of understanding the behavior of masonry assemblies. Consequently, multiple variables influence the parameters of masonry assemblies, which are attributed to the construction process and the age of evaluation [4]. Numerous studies have explored the long-term performance of masonry by investigating various material properties, such as block geometry [5], curing time (*t*) [6], height-to-thickness and length-to-thickness ratios [7], mortar properties [8], and the presence of grouted or un-grouted cells [9]. However, there are limited studies that have investigated the impact of these properties on early-age (i.e., masonry less than 7 days old measured from the point of initial mortar mixing with water) masonry prisms. This paper presents a detailed experimental investigation into the effects of mortar strength gain over time on masonry assembly behavior.

Over the last several years, the development of international standards led to the creation of safe and properly designed, fully cured masonry capable of withstanding lateral loads induced by wind or earthquakes [10,11,12]. However, before masonry is fully cured, it may exhibit only a fraction of its 28 days design strength [13,14]. It was reported that early-age masonry is especially vulnerable to lateral loads during this time because early-age mortar has inconsiderable strength as the hydration phase is not completed during the first stages of construction [15,16]. Based on the news, for example, early-age masonry walls failed due to wind loads during the first stages of construction in Windsor, Ontario, Canada in 2021 and Haines, Florida, United States in 2020, which resulted in the deaths of workers. One potential solution to improve the stability of early-age masonry is the use of temporary bracing, which provides lateral support to masonry walls until they can be connected to the structure and gain sufficient lateral strength as the masonry assembly cures [17].

Two primary resources for designing temporary bracing for masonry include publications by the International Masonry Institute [18] and the Mason Contractors Associations of America [15]. However, a critical examination of these guides revealed their potential inefficiency and ineffectiveness within the design and material requirements [14]. Design codes, such as the National Building Code [19], the masonry construction for buildings code [16], and the design of masonry structures [12], notably lack guidance on loads and material resistances necessary for designing temporary support systems. Consequently, designers rely on engineering judgment, material test data (rather than assembly data), and experience to estimate the reasonable and safe assembly properties of freshly constructed masonry to design temporary bracing [13]. Often, these properties are estimated based on a percentage of the full strength, establishing an empirical relationship between the strength of freshly laid mortar and the strength of hardened mortar. However, since quality assurance testing typically commences after seven days, there is a critical period during construction when designers lack trustworthy data to assess the effectiveness of temporary bracing systems. This lack of data often leads to over-designed temporary bracing, as excessive conservatism is employed to compensate for the lack of understanding.

The compressive properties of early-age masonry are crucial for ensuring structural integrity and safety during construction. Moreover, compressive properties of masonry are some of the crucial parameters in the analysis of masonry walls supported by temporary bracing, such as the numerical evaluation of temporary bracing systems. Both Canadian and American standards focus on the minimum specified compressive strength of masonry units (*f_b_*) and types of mortar to establish the overall masonry compressive strength (*f′_m_*) [11,12,20,21,22]. However, these standards provide field control testing requirements for 7 days and beyond but lack guidelines for the compressive properties of masonry at ages less than 7 days. This study addresses this gap by investigating the average compressive strength of mortar cubes (*σ_mc_*) and masonry prisms (*σ_m_*) during the first 28 days post-construction.

Previous research has extensively examined the compressive properties of masonry constructed with different materials and methods [23]. For instance, a review of the literature on *σ_m_* of masonry constructed with new materials has been conducted, including damage detection methods applied to existing masonry structures [24]. The bond between blocks and mortar was experimentally assessed, considering various mortar types, and prediction models were developed with comparisons to Eurocodes [6,24]. Furthermore, the effects of hollow block properties, grout, and mortar on *σ_m_* were investigated experimentally, and failure modes were categorized [25,26,27].

Additionally, the compressive properties of confined reinforced masonry boundary elements constructed with C-shaped concrete blocks were evaluated, and the relationship between these properties and their structural efficiency was assessed [28]. Studies have also explored the compressive strength of masonry prisms and wallettes, identifying correlations between their compressive strengths numerically [29]. The dependency of *σ_m_* on the size of masonry prisms has been investigated using finite element (FE) simulation, emphasizing the significance of the length-to-thickness and height-to-thickness ratios of the samples [4]. These ratios were compared with international masonry code recommendations, providing suggestions for future revisions. However, limited research has focused on the properties and behavior of early-age masonry, especially *f′_m_*.

Further research has developed empirical formulas to predict *f′_m_* based on extensive databases of compressive test results on masonry prisms, revealing that masonry codes often underestimate *f′_m_* [30]. The influence of joint thickness and *σ_mc_* on the strength and stiffness of masonry made of normal and high-strength concrete blocks has been assessed [31]. Experimental studies have examined the influence of grout and mortar types on *f′_m_*, employing different types of mortar, grout, and concrete blocks [32,33,34]. Constitutive laws using concrete softened theory have been developed to predict the compressive stress–strain (*σ-ε*) behavior of grouted masonry, showing good agreement between experimental and numerical assessments [35]. The effect of alkanolamines on the strength of early-age mortar was studied experimentally recently [36]. The results of the experimental study showed that the compressive strength of 3-day mortar is equal to ~70% of 7-day mortar compressive strength.

The compressive *σ-ε* relationship of hollow concrete block masonry has been evaluated experimentally using various block/mortar combinations. The failure phenomena of mortar joints, blocks, and prisms were investigated using non-contact digital image correlation methods [37]. Numerical relationships between the average secant modulus of elasticity of masonry prisms (*E_m_*) and the corresponding *σ_m_* have been determined. Additionally, new numerical models for the *σ-ε* behavior of hollow concrete block masonry have been developed and compared with existing models in the literature [38]. By analyzing *σ_mc_* and *f_b_*, the behavior of masonry elements such as columns and bridges has been predicted [39,40]. Regression analyses have been conducted to predict the *σ-ε* behavior of unreinforced masonry based on experimental studies of individual masonry elements [41].

The compressive properties of hollow concrete masonry prisms constructed with different types of mortar were studied experimentally. Based on the results, the mortar, governs the masonry failure mechanism [42]. Also, *σ_mc_* was assessed by conducting an experimental study according to various testing methods. The effects of different parameters, including *σ_mc_*, water-to-cement ratio, age of testing, and curing conditions, were investigated on *σ_mc_*. Also, a simplified analytical model was developed, which showed good agreement with the experimental data [43]. It should be noted that *σ_m_* and *σ_mc_* weakly correlate [44] because of the triaxial confinement the unit provides the mortar. Moreover, the compressive properties of different masonry prisms, including couplets and wallettes with different types of masonry units and mortar, were assessed experimentally to investigate the effects of confinement on *σ_mc_* [45]. However, limited research has focused on the properties and behavior of early-age mortar and masonry. 

Dunphy et al. (2021) [14] conducted pioneering investigations into the tensile properties of early-age masonry prisms through a combination of numerical and experimental studies. Their study involved testing two-block prisms at different *t*, 3–72 hours (h), to explore the tensile strength properties of early-age samples. Moreover, they conducted regression analysis to predict the properties of the materials using a limited number of test samples. To address these limitations, Abasi et al. (2023) [13] conducted other experimental and numerical studies for a larger number of early-age masonry prisms and investigated the effects of *t* on the tensile properties of masonry prisms for a wider range of early ages (3–168 h). Moreover, they assessed the relationship between the tensile properties of early-age cubic mortar samples and a wide range of early ages experimentally. According to the regression analysis, some numerical models were developed to predict the tensile properties of both the early-age mortar and masonry prisms against *t*. Furthermore, a micro-modeling FE simulation was developed to predict the tensile behavior of early-age masonry prisms corresponding to *t*, which was not addressed by the experiments [13]. Despite these advancements, most studies on early-age masonry have focused on tensile properties, with limited investigation into compressive properties. The compressive properties of early-age masonry and its relationship with *t* are crucial to assessing the behavior of the early-age masonry subjected to lateral loads.

To address this gap in the literature, this paper focuses on the experimental compressive properties of early-age masonry prisms and mortar cubes. Results of this study can be used for simulation of the materials for numerical assessment of early-age masonry walls supported by temporary bracing subjected to lateral loads, as bracing results in compression stresses in the masonry walls against lateral loads. By continuing this research, the lack of information regarding early-age masonry and the lack of understanding of the design of temporary bracing, especially in Canada, will be addressed. It is anticipated that this research will prepare the necessary compressive design properties, leading to improved guidelines needed to design control devices as well as for construction scheduling (e.g., when precast floor planks can be placed) for concrete-block masonry construction. Regarding the simulation of early-age masonry, both the properties of the ingredients of masonry (such as mortar) and the properties of the masonry assemblages (such as masonry prisms) are necessary. Therefore, early-age cubic mortar samples and masonry prisms are tested to assess the effects of *t* on their compressive properties in this study. After analysis of the data, numerical models are developed to predict the properties against *t*. The results of the tests and the failure modes of the samples are discussed in the following sections. The outline of the paper is as follows. The process and different approaches for the assessment of the compressive properties of masonry are discussed in detail in Section 2. In Section 3, the mortar testing method and its results are illustrated. The compressive properties of masonry prisms are assessed in Section 4. The conclusions are discussed in detail in Section 5.

## 2. Compressive Properties of Masonry

Based on both Canadian [12] and American masonry standards [11], there are two approaches to establishing the design value of *f′_m_* for masonry. The first, and most common method used in Canada is to use Table 3 (clay brick) or Table 4 (concrete block) in CSA-S304 to select *f′_m_* based on *f_b_* and mortar type used in construction. The first method, commonly referred to as the unit strength method, is derived from historical data and does not take mortar strength into consideration. Mortar is specified according to the proportion specifications in CSA-A179, where a minimum compressive strength is not a requirement, and compressive strength tests may be taken for quality assurance but are used only to gauge consistency in the mixture. The second method, which is seldom used in Canadian design, is through experimental testing of masonry prisms to directly establish *f′_m_*. Both methods vary from Canada to the US; however, in both countries, use of the tabular method is more common.

For this study, the experimental method is adopted because the objective is to establish what, if any, relationship there is between *σ_m_* and *t*. To enable the handling of smaller specimens and mitigate damage to freshly laid mortar joints, the test method adopted by the US in ASTM-C1314 is used in this study. Since unit strength is not a variable studied, compressive testing of mortar cubes is conducted to determine what relationship exists between *σ_mc_* and *t*. Test standards for mortar cubes do not vary significantly from Canada to the US, unlike prism testing, and mortar cubes are tested according to the requirements laid out in CSA-A179 and ASTM-C270. In the following sections, the compressive properties of mortar cubes and masonry prisms are discussed, and the results of the corresponding tests are presented.

## 3. Experimental Investigation of Compressive Properties of Early-Age Mortar Cubes

In this section, the compressive properties of early-age mortar cubes are assessed experimentally. Several cubic mortar samples corresponding to different *t* were tested. The results of the tests and the numerical models are presented in the following sub-sections.

### 3.1. Test Setup for Evaluating Compressive Strength of Mortar Cube

**Figure 1:**
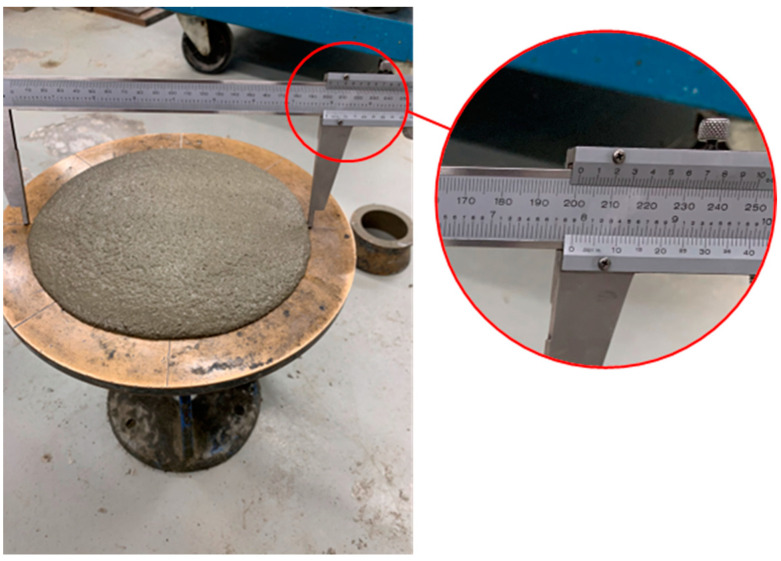
The flow table.

The recommended procedure involved mixing the mortar with a mechanical batch mixer for a duration of 3–5 min (min) [21]. Subsequently, the mortar was to be molded into 50 mm cubes using the molding process outlined in CSA-A3004. The molding process entailed a two-step procedure where a 25 mm thick layer of mortar was placed in the cube during each step. In each step, mortar was tamped 32 times, evenly distributed over the specimen’s surface, and this tamping was carried out in four rounds [46]. Pre-bagged proportionally specified type S-Portland-Lime mortar (in this case, pre-bagged means that the dry ingredients, sand-lime-cement, were proportioned and mixed in a manner where only water was added on the job site) was used as mortar material for all samples in this study to minimize material variability. The volumetric proportions of the pre-bagged S-Portland-Lime mortar is 1:1:3.5–4.5 for Portland cement, lime, and aggregate, respectively [21]. As per CSA-A179, the mortar flow should fall within the range of 110 ± 5% required for laboratory-mixed samples, and this can be determined using the flow table and method outlined in CSA-A3004. Following ten flow tests involving various mixtures of pre-bagged mortar and water, a 7.5:2 volumetric proportion of all dry ingredients (sand–lime–cement) to water resulted in a flow rate of 111%, and this proportion was adopted for the tests in this research. The flow table is represented in Figure 1, and the diameter of the mortar shows the mortar flow. Following the filling of molds, the samples were promptly transferred to a curing room with a humidity level exceeding 90% and a controlled temperature ranging between 20 and 22 °C, where they remained until the scheduled testing time [21]. While CSA-A179 recommends considering a minimum of six samples for fully cured mortar cube compressive testing, this research extended its efforts to conduct an average of 20 tests for each *t* to reduce uncertainty.

**Figure 2:**
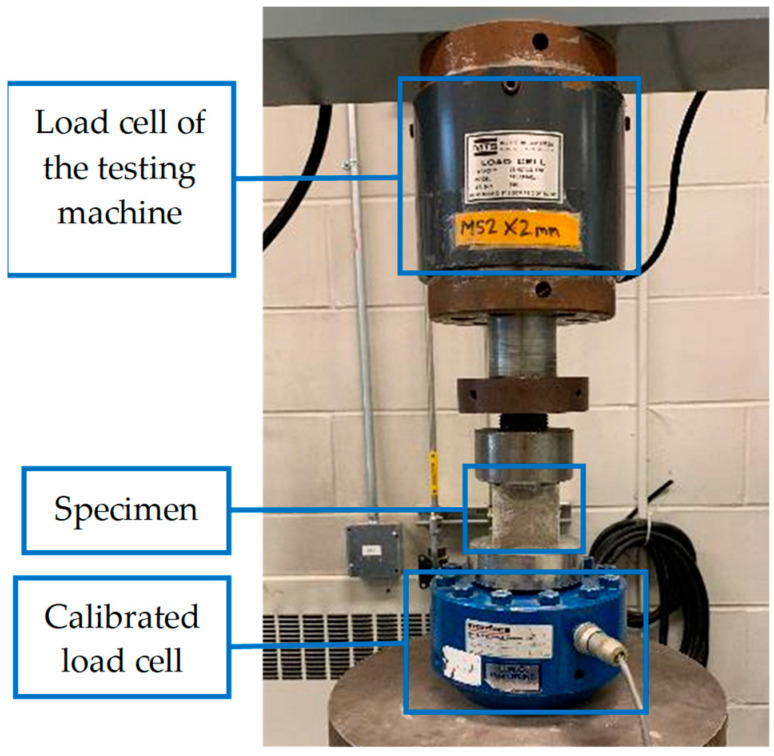
Calibration of the load cell of the mortar cubes testing machine.

The testing apparatus, illustrated in Figure 2, was a displacement control machine comprising two steel plates and a load cell with a substantial capacity of 25,000 kg, significantly exceeding the strength of early-age cubic mortar samples. This machine aligned with the standards for compressive mortar cube testing outlined in ASTM-E4. The loading rate of the test could be at any rate; up to one-half of the expected maximum load may be applied. Then, the remaining load shall be applied at a uniform rate for 20–80 s [21]. In this research, a range of displacement rates was explored to determine the most suitable loading rate, with a final selection of 3 mm/min that adhered to the prescribed guidelines. To calibrate the load cell of the testing machine, another calibrated load cell was added to the machine (attached to the bottom plate of the testing machine), and the specimen was placed between the calibrated load cell and the load cell of the testing machine, as shown in Figure 2 (for just a few samples). Then, the specimen was tested under compression load, and the results obtained from the load cells were compared. As the difference between the results of the load cell was less than 3%, the load cell of the testing machine was calibrated and was suitable to conduct the tests. The mortar samples were tested in the casting direction. The displacement was measured using an embedded Linear Variable Differential Transformer (LVDT), which was a built-in part of the testing machine.

### 3.2. Results and Discussions

The results of a total of 215 tests for 10 groups of *t*, including 3, 4, 6, 13, 18, 24, 48 (2 days), 72 (3 days), 168 (7 days), and 672 h (28 days), are presented below. Based on the logarithmic hydration phase of mortar [13], a higher frequency of intervals was considered in the early stages to account for the logarithmic behavior of mortar. As this study focused on early-age masonry, most of the *t* groups are less than 7 days. However, *t* of 28 days was considered in this study because *t* of 28 days is a reference curing time for some of the design parameters in Canadian masonry design codes [12]. For the analysis of the data, *σ-ε* plot of the samples is provided. As the variation in the data were high, an outlier analysis was conducted to decrease the uncertainty of the results. A value of 50% difference from the average of the data were selected based on CSA-A179-14, which set this threshold as the point where further investigation was required to determine if there were any errors in the mixing or proportioning of a mixture and essentially identified this somewhat as an outlier limit to testing. Although not intended for such early-age samples, it was decided that this was a reasonable and conservative value to adopt for the purposes of this study, where outliers were expected to be more prevalent given the challenges of testing such early-age samples. 

Therefore, data deviating by more than 50% from the average failure stress (*σ_p_*) and the average corresponding strain (*ε_p_*) of all similar-age tests was identified as an outlier and subsequently removed from the dataset. *σ_p_* is the minimum of the maximum compressive stress in the *σ-ε* plot of tests, and the compressive stress corresponds to the strain of 0.03. The strain of 0.03 is considered a threshold based on the engineering judgment that is discussed in the next section. Also, *ε_p_* is the strain corresponding to *σ_p_*. The strain of the samples in these experiments was the ratio of the recorded vertical displacement to the height of the samples. However, as the variation of the data are considerable, with a coefficient of variation (COV) of >30%, even after removing some outliers based on the above-mentioned method, the interquartile range technique is used in this paper as another layer of outlier analysis [47,48]. According to this approach, data exceeding 1.5 times the interquartile range above the third quartile or below the first quartile are classified as outliers. The interquartile range represents the difference between the first and third quartiles. Following the removal of outliers from the dataset in this paper, at least ten valid samples for each *t* are retained for subsequent analysis. After outlier analysis, the COV of *σ_p_* and *ε_p_* regarding all groups of data are <15%.

**Figure 3:**
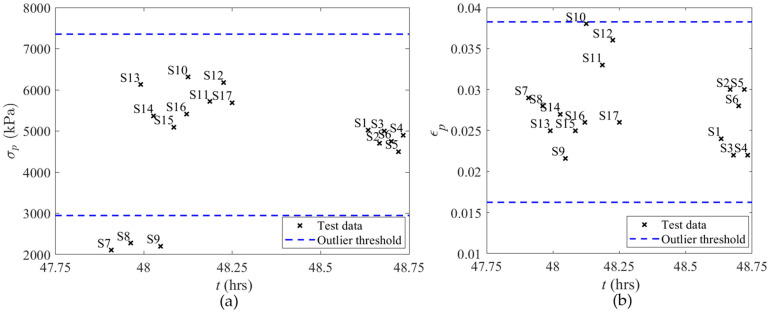
Outlier analysis results for 48 h early-age cubic mortar samples; (**a**) *σ_p_*; (**b**) *ε_p_*.

For instance, the results of the outlier analysis of 48 h samples are presented below. A total of 20 samples were tested for the 48 h group. Before the outlier analysis, the COV of *σ_p_* and *ε_p_* were equal to 39.8% and 16.5%, respectively. Five data points are considered outliers based on the 50% threshold, and these data are removed from the dataset. By using the interquartile range method of outlier analysis, three more samples (S7–S9) are considered outliers, as shown in Figure 3a. Therefore, 12 acceptable samples, with a COV of 7.8% and 10.6% for *σ_p_* and *ε_p_*, respectively, are considered for analysis regarding *t* of 48 h.

**Figure 4:**
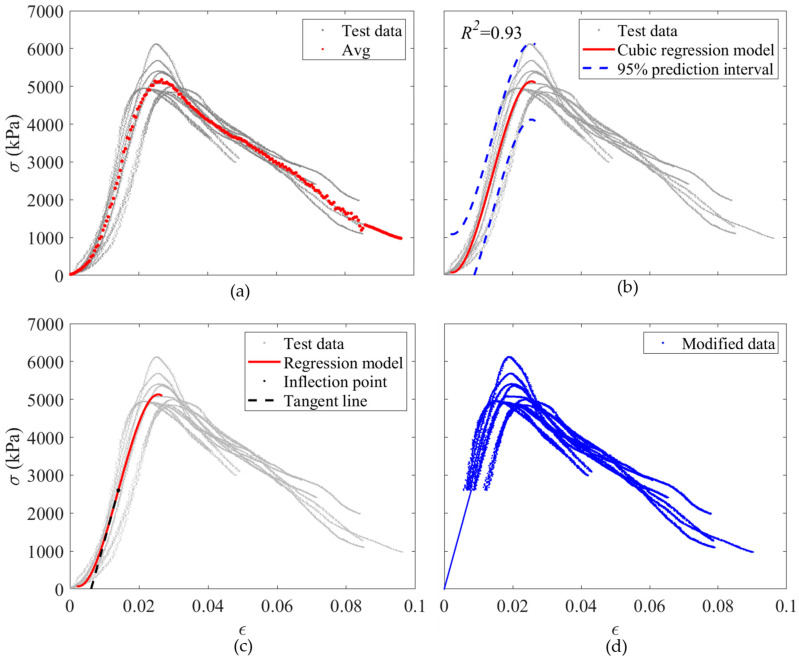
Modification procedure of 48 h cubic mortar samples; (**a**) *σ*-*ε* plot of all acceptable samples and their average; (**b**) The three-degree polynomial regression analysis on the raw data; (**c**) A tangent line intersecting the regression curve precisely at the inflection point; (**d**) Modified data.

Typically, the slope at the initial section of the plot is regarded as the initial modulus of elasticity for the respective sample [49]. Moreover, the slope of the *σ-ε* plot is descending, usually. However, the slope of the *σ-ε* plot of the early-age cubic mortar samples is ascending, as shown in Figure 4a, which can be due to the error of the testing machine and the air bubble between the samples and plates of the machine. Moreover, as the early-age cubic mortar samples are too sensitive, a thin aluminum plate is used to move the samples to the testing position. The small gap between the aluminum plate and the plate of the testing machine can be another reason for this problem. Therefore, the data are modified to remove the ascending-slope part of the *σ-ε* plot before analysis of the data, as described in detail below. Firstly, the average of all *σ-ε* plots of 48 h samples is obtained, as shown in Figure 4a. Then, a regression analysis is conducted to predict the *σ-ε* data before *σ_p_* using a 3-degree polynomial regression model. As shown in Figure 4b, the coefficient of determination, or *R*^2^ value, of the regression model is 0.93, and most of the data points are between the 95% prediction intervals, which shows the robustness of the regression model.

Subsequently, the inflection point of the three-degree polynomial regression model is found, and a tangent line intersecting the regression curve precisely at the inflection point is drawn, as illustrated in Figure 4c for 48 h samples. Then, the raw data before the inflection point is removed and replaced with the tangent line. As a result of this process, the ascending slope part of the *σ-ε* plot is removed, and the modified data represents a typical *σ-ε* plot, which has a descending slope, as shown in Figure 4d. Hence, the slope of the modified data at the start point is identified as the modulus of elasticity. The *ε* of the inflection points calculated based on the above-mentioned method regarding all *t* groups are presented in Table 1. There is not any rational relationship between the *ε* corresponding to the inflection points and *t*, as it occurred due to the error in the testing machine and test setup as explained above.

Regarding the analysis of the *σ-ε* plot of masonry elements, there are different approaches presented in the literature to predict the behavior of masonry, including the polynomial type of constitutive law [50], the fractional type of constitutive law [51], the logarithmic type of constitutive law [38], and the multi-part type of constitutive law [41]. The Priestley-Elder (PE) model is one of the multi-part types of constitutive law, which is one of the common methods for masonry [38]. The PE model includes a parabolic model for the ascending branch of *σ-ε* data and a linear model for the descending branch of *σ-ε* data [52]. Equation (1) represents the PE model, where *ε_p_* is the strain corresponding to the *σ_p_* of the sample, and *A*, *B*, *C*, and *D* are the coefficients of the equations, which can be calculated by regression analysis.

(1) $$\sigma =A\times \varepsilon^{2}+B\times \varepsilon \;0\leq \varepsilon \leq \varepsilon_{p}\;\sigma =C\times \varepsilon +D\;\varepsilon_{p}<\varepsilon$$

Based on the ultimate limit state method design, the maximum usable *ε* for masonry elements is 0.003 [12]. Therefore, the *ε* of early-age masonry is limited to ten times the maximum usable *ε*, which is equal to 0.03 in this paper based on engineering judgment. *σ-ε* plot of samples with *t* ≤ 18 h does not have any peak points, and the ascending branch of the *σ-ε* plot exceeds the *ε* of 0.03. Hence, only the parabolic model is presented for the samples of *t* ≤ 18 h. However, both the parabolic model and linear model (regarding the ascending and descending parts of the *σ-ε* plot, respectively) are presented for the samples older than 18 h.

**Figure 5:**
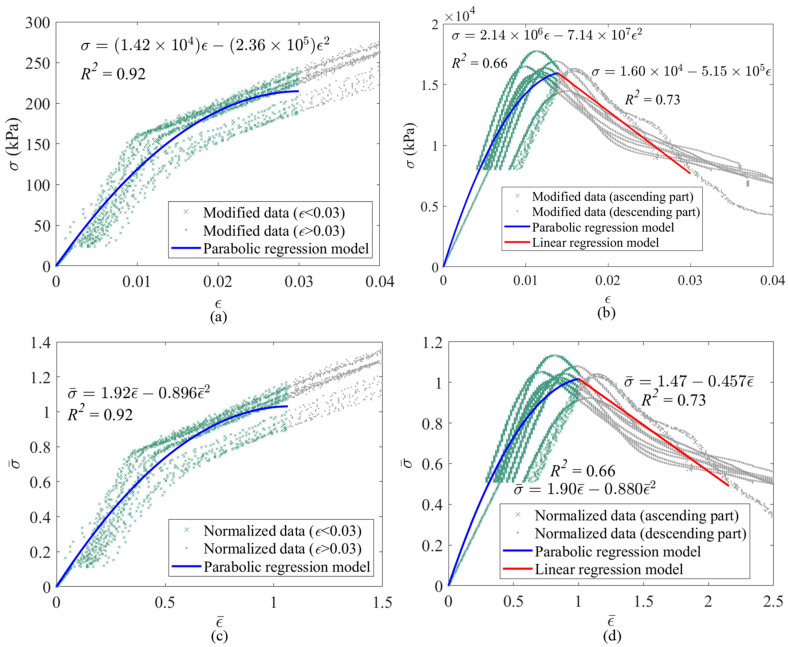
PE regression analysis on mortar cubes testing; (**a**) 13 h (unnormalized data); (**b**) 168 h (unnormalized data); (**c**) 13 h (normalized data); (**d**) 168 h (normalized data).

After the above-mentioned pre-analysis of the data, *σ_mc_* is used instead of *σ_p_* because the average of *σ-ε* plots of tests is used for analysis. For instance, *σ-ε* plots as well as the PE model of *t* of 13 h (as an example of *t* ≤ 18 h groups) and *t* of 168 h (as an example of *t* > 18 h groups) are presented in Figure 5. As shown in Figure 5a, there is no peak point in the *σ-ε* plot of 13 h samples, and just the parabolic section of the PE model is presented for these data. Moreover, the relationship between *σ* and *ε* and the *R*^2^ value of the parabolic regression model of 13 h samples (which is equal to 0.92) is presented in Figure 5a. However, Figure 5b proves that they both are parabolic, and the linear part of the PE model can be presented for samples older than 18 h (such as 7 days). The equations of parabolic and linear regression models and *R*^2^ values (which are equal to 0.66 and 0.73, respectively) are shown in Figure 5b.

To simplify the equations of the regression models, the data are normalized. Therefore, the data are divided by *σ_mc_* and the strain corresponding to *σ_mc_* (*ε_mc_*). However, as there is not any peak point in the average *σ-ε* plot of samples earlier than 18 h, it is assumed that *ε_mc_* and *σ_mc_* represent the *ε* of 0.03 and its corresponding *σ*, respectively. Equation (2) shows the normalized stress ($\bar{\sigma })$ and normalized strain $\left(\bar{\varepsilon }\right)$ equations. Moreover, Equation (3) presents the PE model equations based on the normalized data. The equations of both parts of the PE model for 13 and 168 h samples are presented in Figure 5c,d, respectively, based on the normalized data. By comparing the equations of the regression models presented in Figure 5a–d, it can be mentioned that normalizing the data can simplify the equations considerably.

(2) $$\bar{\sigma }=\sigma /\sigma_{mc}\;\bar{\varepsilon }=\varepsilon /\varepsilon_{mc}$$

(3) $$\bar{\sigma }=a\times {\bar{\varepsilon }}^{2}+b\times \bar{\varepsilon }\;(0\leq \bar{\varepsilon }\leq 1)\;\bar{\sigma }=c\times \bar{\varepsilon }+d\;(1<\bar{\varepsilon })$$

The results of the analysis of the test data regarding all *t* groups are presented in Table 2. It should also be noted that *ε* calculated in mortar cube compression tests is an engineering strain. It means that a constant cross-sectional area is assumed in this analysis. However, the cross-section increases with vertical load due to Poisson’s effects. Therefore, assuming a constant cross-sectional area results in some levels of error near *σ_mc_* because of the high levels of vertical strain in early-age specimens. As shown in Table 2, at least ten acceptable tests are considered for each *t* group, and more than 12 tests are accepted for most *t* groups. As shown, the average initial modulus of elasticity of mortar cubes (*E_mc_*) and *σ_mc_* increase logarithmically as *t* increases, which shows the importance of *t*. It should be noted that *E_mc_* and *σ_mc_* are the average of the corresponding parameters regarding all test data for each *t* group. All coefficients, including *a*, *b*, *c*, and *d*, are presented, and the corresponding equations regarding the regression models, developed based on all test data regarding each *t* group, can be calculated using Equations (2) and (3). Most of the *R*^2^ values of the regression models are more than 60%, which shows the robustness of the models. *σ_mc_* of early-age mortar cubes earlier than 24 h is considerably less than the strength of fully cured samples. For example, the ratios of the *σ_mc_* of 6 and 24 h samples to the strength of 672 h samples are 0.001 and 0.039, respectively.

**Figure 6:**
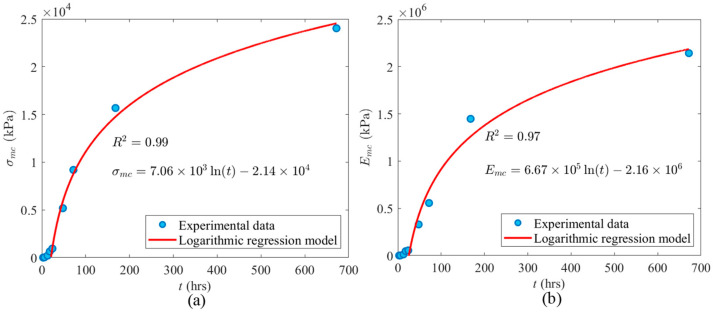
Logarithmic regression analysis regarding the compressive properties of early-age mortar cubes against *t*; (**a**) *σ_mc_*; (**b**) *E_mc_*.

For instance, in order to derive the equations depicting the relationship between *σ* and *ε* for the 168 h samples, Equation (2) should be transformed into Equation (4) by substituting the values of *σ_mc_* and *ε_mc_* specific to the 168 h samples. As indicated in Table 2, *σ_mc_* and *ε_mc_* of 168 h samples are equal to 1.56 × 10^4^ kPa and 0.014, respectively. Subsequently, by incorporating Equation (4) into Equation (3), Equation (5) takes shape as follows. Referring to Table 2, the coefficients of *a*, *b*, *c*, and *d* for the 168 h samples are determined as −0.880, 1.90, −0.457, and 1.47, respectively. Equation (5) presents the *σ-ε* relationship for the 168 h samples. According to *σ_mc_* and *E_mc_* presented in Table 2 for all *t* groups, a logarithmic regression analysis is conducted to predict *σ_mc_* and *E_mc_* of new *t* groups, which are not addressed in the experimental study. Figure 6a,b show the regression models for *σ_mc_* and *E_mc_* against *t*, respectively. Equations (6) and (7) can predict *σ_mc_* and *E_mc_* against *t*. The *R*^2^ values of the equations of *σ_mc_* and *E_mc_* are equal to 0.99 and 0.97, which shows the accuracy of the regression models. For example, *σ_mc_* and *E_mc_* of a 120 h sample can be predicted as 1.24 × 10^4^ kPa and 5.35 × 10^6^ kPa using Equation (6) and Equation (7), respectively.

(4) $$\bar{\sigma }=\frac{\sigma }{1.56\times 10^{+4}};\;\bar{\varepsilon }=\frac{\varepsilon }{0.014}$$

(5) $$\sigma =\left(-7.14\times 10^{+7}\right)\times \varepsilon^{2}+(2.14\times 10^{+6})\times \varepsilon \;\;\;\;\;\;\;\;\;\;0\leq \varepsilon \leq 0.014\;\sigma =\left(-5.15\times 10^{+5}\right)\times \varepsilon +(1.60\times 10^{+4})\;\;\;\;\;\;\;\;\;\;\;\;\;\;\;\;\;\;\;\;\;\;0.014<\varepsilon$$

(6) $$\sigma_{mc}\left(t\right)=\left(7.06\times 10^{+3}\right)\times \ln\left(t\right)-(2.14\times 10^{+4})$$

(7) $$E_{mc}\left(t\right)=\left(6.67\times 10^{+5}\right)\times \ln\left(t\right)+(2.16\times 10^{+6})$$

**Figure 7:**
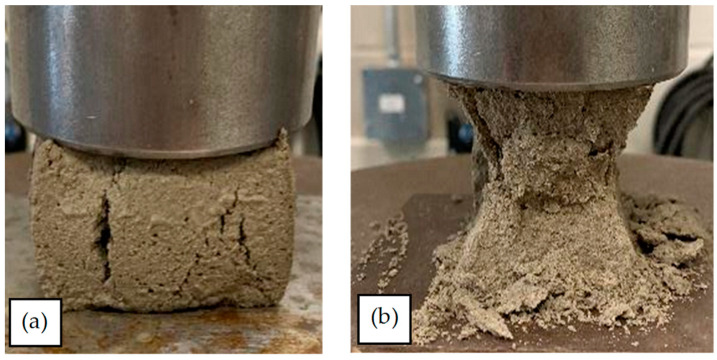
Failure modes of mortar cubes; (**a**) 3 h; (**b**) 24 h.

As shown in Figure 6a,b, the regression models for *σ_mc_* and *E_mc_* against *t* do not intersect the origin. *σ_mc_* and *E_mc_* of mortar cubes earlier than 20.8 h is considerably less than *σ_mc_* and *E_mc_* of fully cured samples. The ratios of the *σ_mc_* and *E_mc_* of 24 h samples to the strength of 672 h samples are 3.95% and 2.55%, respectively. Therefore, the regression models intersect the *x*-axis of the plots at *t* = 20.8 h. The reason behind this is that *t* = 20.8 h coincides with the end of the dormant (or induction) phase of hydration in the cement paste in the mortar. When *t* < 20.8 h, the cement in the mortar has not yet undergone its primary hydration phase; after the final set, when the cement in the mortar undergoes its dormant phase of hydration but before it enters its main phase, there is no cohesion to the mortar itself. This is visually evident in the appearance of the failure of the mortar cube samples shown in Figure 7. As indicated in Figure 7a, before the main phase of hydration, the mortar is plastic and is simply pushed or squeezed between the platens of the test machine. However, when *t* > 20.8, the main phase of hydration is initiated in the cement, and the failure mode of the cube begins to take the shape of the commonly known conical shear pattern associated with concrete testing, as shown in Figure 7b. It is consistent with the findings reported in the literature, as the maximum heat of hydration occurs at ~21.8 h after the mixture of mortar with water in average regarding different types of mortar [53]. In the main phase of hydration, alite and belite start to react and form calcium silicate hydrate and calcium hydroxide. This makes sense because the bonds between these internal chemicals start to form, which is why the cubes begin to fail in the more common conical shear failure pattern [54].

## 4. Experimental Investigation of Compressive Properties of Early-Age Masonry Prisms

In this section, the compressive properties of early-age masonry prisms are discussed. Two-course concrete block prisms are tested to evaluate the relationship between the compressive properties of early-age masonry and *t*. The details of the test process and the results are presented in the following sub-sections.

### 4.1. Test Setup for Comprehensive Tests of Masonry Prisms

Testing of mortar within a masonry assembly is required to understand the compressive properties of masonry under in-wall conditions [55]. In previous research, uniaxial tensile tests were conducted with both isolated mortar samples and mortar applied to masonry prisms [13]. Testing requirements for concrete block masonry prisms are specified by Annex D in CSA S304 and ASTM-C1314, as referenced by TMS 402/602. Although they are similar in approach, there are several notable differences between these standards in their approach to establishing *f′_m_*. Of relevance to this research program is the fact that ASTM-C1314 permits the testing of saw-cut units 2 courses in height. This is deemed a necessary feature to ensure the safe handling of the specimens. Due to the time-sensitive nature of these tests, a modified approach to testing was adopted here, similar to the modified testing regime used for direct tension [13], which is detailed below. The masonry prisms were not capped in this research.

**Figure 8:**
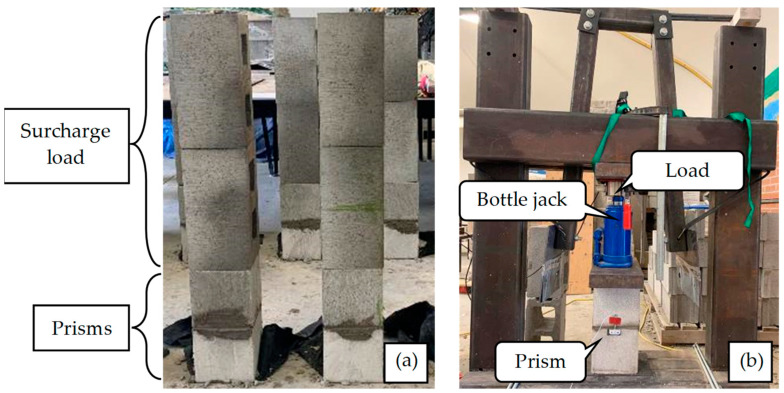
Masonry prism testing; (**a**) Curing process and condition; (**b**) Test setup.

In this research, a modified ASTM-C1314 prism was adopted using a saw-cut half unit (containing one cell). Moreover, this research focused on masonry constructed at fields, not masonry constructed using pre-cast prisms. The temperature and humidity were monitored on construction and test days, and the average temperature and humidity were around 16 °C and 35%, respectively, satisfying ASTM-C1314 requirements. Due to the nature of the specimens and their age at testing, moisture tight bags were not used during curing. After each specimen was constructed, two full blocks were put on the prisms endwise, as depicted in Figure 8a, to provide a surcharge load that would be expected during construction and to follow the procedure adopted with direct tension tests [13]. Regarding the loading rate of the machine, up to one-half of the expected maximum load, the loading rate can be at any convenient rate. However, after that point, the load should be applied at a uniform rate in not less than one min nor more than two min [12,56]. To determine *E_m_*, the average value of the secant modulus of at least five prisms should be calculated. *E_m_* is measured over a *σ* extending from 0.05 to 0.33 of the measured *σ_m_* [12]. As shown in Figure 8b, the test setup included a steel frame, a load cell, a bottle jack, and two steel plates. The load cell had a capacity and accuracy of 6.80 × 10^2^ kg and ±1%, respectively. After putting the prisms under the frame, the load cell was placed between the prisms and the frame. Two plates were placed between the load cell and prisms, as well as the load cell and the frame, to distribute the load uniformly (see Figure 8b). Then, using the handle of the load cell, the load was increased gradually until prism failure.

**Figure 9:**
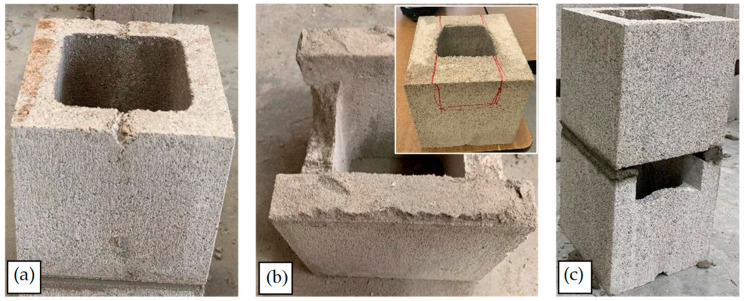
Blocks and prisms: (**a**) intact one-core block; (**b**) sawn one-core block; (**c**) the two-block prism.

The same mortar as used in mortar cube tests was mixed to the same consistency with a 4:1 volumetric proportion of the pre-bagged dry ingredients (sand-lime-cement) to water. The concrete masonry blocks used in this research had a minimum *f_b_* of 15 MPa, a maximum water absorption of 175 kg/m^3^, and a minimum density of 2 × 10^3^ kg/m^3^ [57]. Initially, prisms were constructed with full-concrete blocks (390 × 190 × 190 mm) and half-concrete blocks (190 × 190 × 190 mm) mortared face shells and webs according to the requirements of ASTM-C1314. However, the strength of these prisms exceeded the capacity of the load cell without any failure initiating in the prism. To correct for this, face shell bedded mortar was instead adopted based on the requirements of CSA-S304, and webs were saw-cut to ensure control in the precise area of each unit that is to be loaded, as shown in Figure 9b. The remainder of the prisms were all constructed in this fashion, as illustrated in Figure 9c. To obtain the exact size of the prisms, the length and width at the edges of the top and bottom faces of the prisms were measured to the nearest 1 mm. Then, the average length and width of the four measurements of each dimension were considered for the calculation of *σ_m_*. Also, the height of the prism at the center of each face was measured to the nearest 1 mm, and the average of the four measurements was considered the height of the prisms [56]. In this research, the length and width of prisms were equal to 190 mm, with their height equal to 390 mm. The effective mortared face shell thickness of prisms was equal to 36.2 mm [44]. Therefore, the effective area between the blocks, subjected to the compression force, was equal to 1.37 × 10^4^ mm^2^.

**Figure 10:**
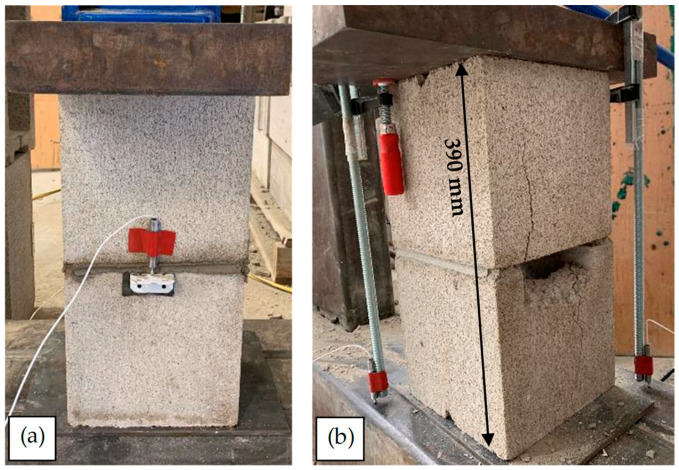
LVDT placement in masonry prism testing; (**a**) monitoring mortar layer; (**b**) monitoring the entire length of the sample.

Four LVDT sensors were attached to all sides of the prisms to monitor the vertical displacement during the tests to ensure a concentric response in the prism to the load. The LVDTs had a capacity and accuracy of 12 mm and ±2%, respectively, and no prisms were observed to undergo an eccentric response to loading. The hydraulic jack had a capacity of 3.00 × 10^4^ kg. As the strength of the early-age mortar was considerably less than the strength of blocks, it was predicted that most of the vertical displacement in early-age prisms, unlike fully cured samples, would happen in the mortar joint. Therefore, two different arrangements of sensors were used. Some specimens had LVDTs located only at the mortar joint itself, as indicated in Figure 10a. For other specimens, the displacement of the entire height of the prism was monitored to permit comparison between the two. In these specimens, as shown in Figure 10b, C-clamps were attached to the top of the steel plate of the setup, and the LVDTs were attached to the rod of the C-clamps. As the difference in the results was considerable (more than 40%), the LVDTs monitored the whole length of the samples for the rest of the tests (see Figure 10b). The reason for this observation is discussed in the following section.

### 4.2. Results and Discussions

In total, 32 prisms were tested during this study. Four groups of *t*, including 6, 18, 24, and 168 h (7 days), were considered to assess the effects of *t* on the compressive properties of early-age masonry prisms. A total of 11 prisms were constructed with full mortar bedding, and an additional 21 were constructed with face shell bedding only. Due to the testing issues with the full bed prisms, samples tested at *t* of 24 h were omitted from analysis due to the inconsistent ability of the testing machine to successfully cause failure. Overall, there was a good agreement between peak *σ* of the intact prisms and sawn-cut prisms, so the results of both are presented below together.

**Figure 11:**
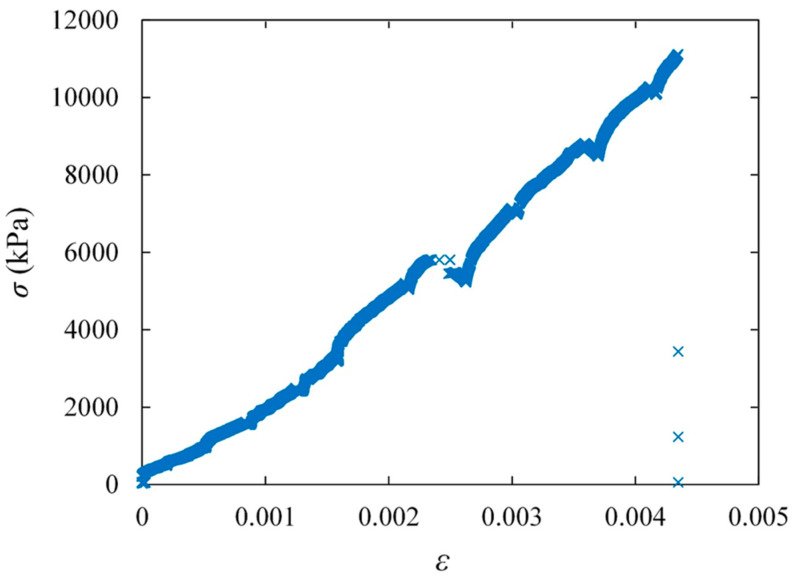
The *σ-ε* plot of a 6 h masonry prism subjected to compression load.

At least 10 prisms were tested from each of the 6 and 168 h groups; however, it was decided to test only five prisms for the 18 h group because the evaluated COV of *σ_m_* of the samples resulted in less than 15%. An outlier analysis (interquartile range technique) was conducted to decrease the variation in the data and find a meaningful correlation between *t* and *σ_m_*. Therefore, at least 10 acceptable samples for each 6 and 168 h group and at least five acceptable samples for the 18 h group are analyzed, and the results are presented below. The *σ-ε* plot of a 6 h prism is shown in Figure 11 as an example of the data collected, which shows the *σ-ε* of the mortar joint. As shown, the compressive response of the six-hour prism is linear until the abrupt failure of the blocks at the end of the test. Therefore, there is not any clear failure point in the plot regarding the early-age mortar. Hence, for all *t* groups, tests are stopped after the complete failure of the prisms (failure of both mortar and blocks). The fluctuations in the force data occurred due to the inconsistent loading rate, as a hydraulic-manual bottle jack was used in the setup. Despite every effort made to maintain a consistent loading rate, fluctuations in the loading rate were inherently unavoidable.

The test results of the masonry prism testing are presented in Table 3. The maximum COV regarding *t* is 6.6%, corresponding to the 6 h group. As shown, there is a good agreement among *σ_m_* of different *t.* For instance, the *σ_m_* of the 6 h prisms is just 0.6% different from the *σ_m_* of the 7 day prisms. In this case, *σ_m_* shows the maximum compressive stress applied to masonry prisms before collapse of the prisms (failure of both mortar and blocks). Although there is not a considerable difference among *σ_m_* of prisms, the vertical displacement of prisms corresponding to *σ_m_* (*δ_m_*) is considerably different among various *t* groups. For example, *δ_m_* of 6 and 18 h samples are 8.5 and 7.8 times larger than *δ_m_* of 7 days samples, respectively. Hence, although the early-age mortar compressed significantly, it is clear that the failure ultimately comes to the block in the form of a tensile splitting arising from the lateral expansion of the mortar when in the triaxial state.

Therefore, from a life safety perspective, the compressive failure load for masonry prisms seems to be independent from *t*. This can be attributed to the triaxial confinement of the mortar in a face shell and the loss of plasticity due to free water loss from absorption by the masonry unit. It should be noted that *σ_m_* and *σ_mc_* are not strongly comparable [44] because of this triaxial confinement. However, failure in masonry prism testing should also be limited by a displacement threshold (in addition to the crashing of the unit/prism), since it is not practical to have mortar squeezed out of a joint. As shown, the early-age specimens indicate that the early-age mortar undergoes a significantly higher level of deformation.

This considerable *δ_m_* can be attributed to the hydration phase of mortar, which was discussed in Section 3.2. As evaluated, when *t* < 20.8 h, the cement in the mortar has not yet undergone its primary hydration phase, and there is no cohesion to the mortar itself. However, unlike mortar cubes, prisms seemed to show some levels of cohesion when *t* < 20.8, and it is speculated that the triaxial confinement of the mortar provides resistance to enable failure to be dictated by the unit. As shown in Table 3, the strain corresponding to the *σ_m_* (*ε_m_*) of 7 days samples is equal to 0.003, which matches the maximum usable *ε* for masonry elements in the ultimate limit state method design [12]. *ε_m_* of 6 and 18 h samples is not calculated because the displacement of the whole height of prisms is monitored in this study; however, most of the displacement has happened in the mortar joint in early-age prisms (such as 6 and 18 h samples). 

Therefore, two displacement thresholds, including 1.17 and 3 mm, are considered to evaluate how much compressive load an early-age prism could tolerate without being detrimental to its appearance/long-term performance because of considerable deformation. The displacement threshold of 1.17 mm corresponds to *ε* of 0.003, the maximum usable *ε* for masonry elements in the ultimate limit state method design [12]. Moreover, the displacement threshold of 3 mm is equal to the maximum tolerance of construction for a mortar joint in masonry [16]. Regarding the displacement of 1.17 mm, the ratio of *σ* tolerated by 6 and 18 h samples to *σ* tolerated by 7 days samples is 3% and 13%, respectively. Therefore, the early-age masonry prisms can be subjected to limited *σ* by considering a 1.17 mm displacement threshold. It shows the effects of the hydration phase on the cohesion of mortar (as discussed in previous sections in detail) and the performance of masonry prisms. At the same time, *σ* corresponding to the displacement of 1.17 mm tolerated by early-age masonry prisms is considerably greater than the *σ_mc_* of mortar cubes at the same age (see Figure 6), which proves the triaxial confinement of the mortar.

**Figure 12:**
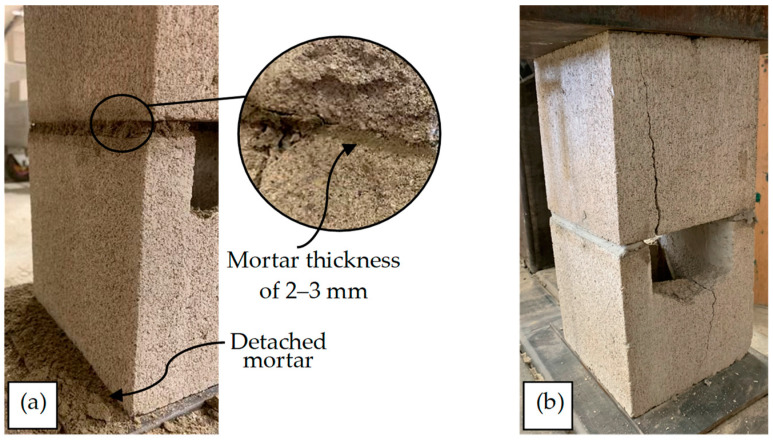
The failure mode of masonry prisms; (**a**) 6 and 18 h samples; (**b**) 168 h samples.

To elaborate on the previous paragraph and the compressive behavior of the early-age masonry prisms, the failure modes of the prisms are presented below. According to Figure 12a, during the compressive tests on the early-age masonry prisms (6 and 18 h), the mortar bed is subjected to significant compression, resulting in its squeezing, reduction in thickness, and subsequent detachment from the prisms. The thickness of the mortar bed of some 6 h prisms was measured during the tests, and it was ~10 mm and ~2–3 mm before and after the tests, respectively, as shown in Figure 12a. This deformation and detachment happen gradually during the tests, and there is not a clear, abrupt failure point for the mortar bed. Therefore, it was not possible to monitor *σ* corresponding to the start of deformation and detachment of the mortar. Figure 12b shows the failure mode of a 168 h prism. As shown, there was not any considerable displacement in the mortar before the failure of the blocks. Therefore, the failure mode of masonry prisms regarding *t* ≤ 20.8 h, when the primary hydration phase has not started yet, is determined by the failure of mortar, which is deformation and detachment. However, the failure mode of prisms depends on the failure mode of blocks when *t* > 20.8 h, as described below.

**Figure 13:**
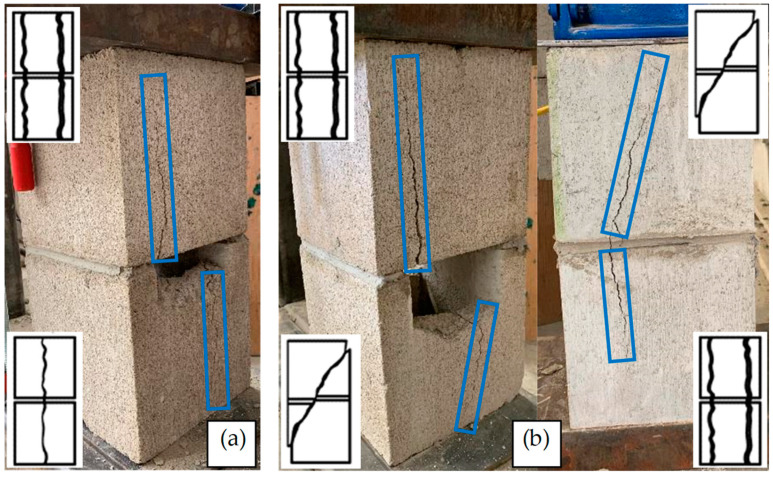
The failure modes of the masonry prisms; (**a**) face–shell separation; (**b**) combination of shear break and face shell separation.

ASTM-C1314 considers seven failure modes for blocks in masonry testing, including conical break, cone and shear, cone and split, tension break, semi-conical break, shear break, and face–shell separation. Two types of the above-mentioned failure modes, including face shell separation and shear break, have happened in the tests. As shown in Figure 13a, both blocks have a face shell separation failure mode in some of the tests. However, in some cases, the failure modes of the top and bottom blocks are different, as presented in Figure 13b. In these cases, one of the blocks has face shell separation, and the other block has a shear break. The sketches of the relative mode failures are shown in Figure 13 based on ASTM-C1314.

## 5. Conclusions

In this paper, the compressive properties of early-age mortar cubes and masonry prisms against *t* were assessed experimentally. It is anticipated that this research will prepare the necessary compressive design properties, leading to improved guidelines needed to design control devices (e.g., temporary bracing) as well as for construction scheduling (e.g., when precast floor planks can be placed) for concrete-block masonry construction. The following conclusions were drawn from the proposed study:

- The variation in the results of early-age mortar cubes and masonry prism testing was considerable. Therefore, the number of tests should be greater than the minimum number of tests suggested in masonry codes. For example, CSA-A179 recommends a minimum of six samples for fully cured mortar cube compressive testing; however, this study suggested an average of 20 tests for each *t* to minimize COV (less than 20–25%). Moreover, the threshold of 50% difference from the average of the data, suggested in masonry codes, was not enough for the analysis of early-age masonry data. Therefore, outlier analysis was necessary, as it reduced the COV of some groups of data by over 80%.
- Based on regression analysis, the *σ-ε* behavior of mortar cubes regarding all *t* groups, as well as the regression models for *σ_mc_* and *E_mc_* against *t* can be predicted for any *t* groups, which were even not addressed in the experimental study, with *R*^2^ of more than 0.95. *σ-ε* plot of *t* ≤ 18 h samples does not have any peak points. Therefore, this study suggested a threshold of 0.03 for *ε* as the failure of samples by adjusting the maximum usable *ε* for masonry elements in the ultimate limit state design method presented in masonry design codes. Moreover, the PE model was edited based on the output of this research. Only the parabolic part of the PE model should be used for the samples with *t* ≤ 18 h. However, the original PE model, including both parabolic and linear parts (regarding the ascending and descending parts of the *σ-ε* plot, respectively), can be applied for the analysis of samples older than 18 h.
- *E_mc_* and *σ_mc_* increased logarithmically as *t* increased, and the developed regression models did not intersect the origin because mortar cubes tested at ages less than 20.8 h had not yet undergone their primary hydration phase and there was no cohesion to the mortar itself. In this case, the failure mode was more akin to a soil failure than the shear compression failure (conical shear pattern) associated with fully cured cementitious materials. For example, 24 h mortar obtains only ~5% of *σ_mc_* of fully cured mortar. However, the failure mode of older mortar cubes (*t* > 20.8 h) was like the failure mode of fully cured mortar or concrete (conical shear failure mode) because of the formation of calcium silicate hydrate and calcium hydroxide.
- Like mortar cubes, the failure mode of masonry prisms depends on the hydration phase of the mortar. The failure mode of masonry prisms regarding *t* ≤ 20.8 h, when the primary hydration phase has not started yet, is determined by the failure of mortar, which is deformation and detachment. However, the failure mode of prisms after the start of the hydration phase (*t* > 20.8 h) depends on the failure mode of blocks. Based on the results, concrete blocks follow the failure modes presented in ASTM-C1314, including conical break, cone and shear, cone and split, tension break, semi-conical break, shear break, and face shell separation.
- Regarding the performance perspective, there was a practical limit to the compressive loads that could be resisted by the early-age masonry without detracting from the appearance and excessive smooshing of the mortar joint, which of course would be qualitative. For example, 18 h samples obtained only ~13% of their full compressive strength. However, regarding the life safety perspective, the compressive failure load for masonry prisms was independent from *t*.

There are different types of mortar and blocks in the industry. As it was not possible to use all types of materials in experiments, a common type of mortar and blocks in Canada were used for experiments, which could be considered an assumption for this research. Moreover, only two-block prisms were studied in this paper. In future work, more course prisms (such as three- and five-block prisms) can be assessed.

## Figures and Tables

**Table 1:** *ε* corresponding to the inflection points of early-age mortar cubes testing.

*t* (h)	*ε* Corresponding to the Inflection Points
3	0.005
4	0.007
6	0.014
13	0.009
18	0.010
24	0.015
48	0.014
72	0.017
168	0.012
672	0.0126

**Table 2:** Results of analysis of compressive early-age mortar cubes testing.

*t* (h)	*N*	*E_mc_* (kPa)	*σ_mc_* (kPa)	*ε_mc_*	*a*	*b*	*c*	*d*	*R*^2^ (Cubic)	*R*^2^ (Linear)
3	16	1.96 × 10^2^	4.96	0.030	−0.911	1.58	-	-	0.63	-
4	13	3.16 × 10^2^	6.89	0.030	−0.995	1.36	-	-	0.61	-
6	15	1.46 × 10^3^	3.22 × 10^1^	0.030	−0.684	1.67	-	-	0.75	-
13	11	1.21 × 10^4^	2.08 × 10^2^	0.030	−0.896	1.92	-	-	0.92	-
18	15	4.66 × 10^4^	6.45 × 10^2^	0.029	−1.58	2.48	-	-	0.64	-
24	11	5.45 × 10^4^	9.48 × 10^2^	0.024	−1.13	2.10	−0.064	1.03	0.62	0.53
48	12	3.29 × 10^5^	5.18 × 10^3^	0.021	−0.847	1.85	−0.314	1.32	0.72	0.57
72	14	5.56 × 10^5^	9.19 × 10^3^	0.017	−0.628	1.66	−0.296	1.33	0.61	0.49
168	10	1.45 × 10^6^	1.56 × 10^4^	0.014	−0.880	1.90	−0.457	1.47	0.66	0.73
672	11	2.14 × 10^6^	2.40 × 10^4^	0.012	−0.506	1.53	−0.483	1.51	0.84	0.71

**Table 3:** The results of compressive masonry prism testing.

*t* (h) (COV%)	*N*	*σ_m_* * (kPa) (COV%)	*δ_m_* ** (mm) (COV%)	*ε_m_* (COV%)	*σ* Corresponding to Displacement of 1.17 mm (kPa)	*σ* Corresponding to Displacement of 3 mm (kPa)
6 (6.6%)	10	1.16 × 10^4^ (8.7%)	1.15 × 10^1^ (19.3%)	-	3.81 × 10^2^	1.27 × 10^3^
18 (4.6%)	5	1.10 × 10^4^ (8.1%)	1.07 × 10^1^ (15.3%)	-	1.60 × 10^3^	4.10 × 10^3^
168 (0.5%)	10	1.15 × 10^4^ (13.2%)	1.36 (29.4%)	0.003 (29.4%)	1.15 × 10^4^	-

* The effective face shell area was considered to calculate *σ_m_*. ** *δ_m_* is the vertical displacement of prisms corresponding to *σ_m_*.

## Data Availability

The original contributions presented in the study are included in the article, further inquiries can be directed to the corresponding author.
